# Insights into Cardiac IKs (KCNQ1/KCNE1) Channels Regulation

**DOI:** 10.3390/ijms21249440

**Published:** 2020-12-11

**Authors:** Xiaoan Wu, H. Peter Larsson

**Affiliations:** Department of Physiology and Biophysics, Miller School of Medicine, University of Miami, Miami, FL 33136, USA; xxw320@miami.edu

**Keywords:** long QT syndrome, IKs, KCNQ1, KCNE1, PUFA, Kv channel, PIP_2_, cardiac arrhythmias, PKA, ATP

## Abstract

The delayed rectifier potassium IKs channel is an important regulator of the duration of the ventricular action potential. Hundreds of mutations in the genes (*KCNQ1* and *KCNE1*) encoding the IKs channel cause long QT syndrome (LQTS). LQTS is a heart disorder that can lead to severe cardiac arrhythmias and sudden cardiac death. A better understanding of the IKs channel (here called the KCNQ1/KCNE1 channel) properties and activities is of great importance to find the causes of LQTS and thus potentially treat LQTS. The KCNQ1/KCNE1 channel belongs to the superfamily of voltage-gated potassium channels. The KCNQ1/KCNE1 channel consists of both the pore-forming subunit KCNQ1 and the modulatory subunit KCNE1. KCNE1 regulates the function of the KCNQ1 channel in several ways. This review aims to describe the current structural and functional knowledge about the cardiac KCNQ1/KCNE1 channel. In addition, we focus on the modulation of the KCNQ1/KCNE1 channel and its potential as a target therapeutic of LQTS.

## 1. Introduction

The cardiac IKs (KCNQ1/KCNE1) channel is one of the main contributors to the repolarizing currents that regulate the ventricular action potential duration (APD) and thus the QT interval in the electrocardiogram [1,2,3]. Mutations in cardiac KCNQ1/KCNE1 channels are the most common cause of congenital defects that cause long QT syndrome (LQTS) [4,5,6]. LQTS is a heart disorder that causes cardiac arrhythmias and 3000 to 4000 sudden deaths in children and young adults in the USA each year [4,6]. Mutations of KCNQ1/KCNE1 channels that reduce IKs currents prolong the QT interval by lengthening the duration of the ventricular action potential [3]. The KCNQ1/KCNE1 channel has been proposed as a potential target for the development of LQTS treatment [7]. One study [8] suggested that the repolarization reserve of KCNQ1/KCNE1 channels is important to prevent the development of ischemia- and reperfusion-induced arrhythmias. The same study showed that the KCNQ1 protein was downregulated in response to ischemia. Therefore, a reduction in KCNQ1/KCNE1 might play a similar role in developing arrhythmia in both congenital LQTS and ischemia- and reperfusion-induced arrhythmias.

The KCNQ1/KCNE1 channel is a member of the super family of voltage-gated ion channels. Voltage-gated ion channels are a class of transmembrane proteins that are activated by membrane potential and play a crucial role in regulating cellular excitation in diverse cell types including cardiomyocytes and neurons. For example, in cardiomyocytes, voltage-gated Na^+^ (Nav), K^+^ (Kv) and Ca^2+^ (Cav) channels are necessary for the initiation, maintenance, propagation and termination of action potentials [1,3,9].

The KCNQ1/KCNE1 channel consists of the alpha-subunit KCNQ1 and the beta-subunit KCNE1 [10,11]. Four KCNQ1 subunits form a Kv channel that is modulated by 1–4 KCNE1 subunits [12,13]. KCNE1 modulation is crucial for the KCNQ1/KCNE1 channel function, but the mechanisms by which KCNE1 interacts with KCNQ1 and thus modulates KCNQ1 are not fully clear yet. Like other Kv channels, the KCNQ1/KCNE1 channel contains both a voltage-sensing domain (VSD) and a pore domain (PD) [14]. Upon activation, the positively charged voltage sensor of VSD senses the membrane potential change and moves outwards within the membrane, opening the pore through the VSD–PD coupling [15,16,17]. Several modulators can regulate the KCNQ1/KCNE1 activity. They can either activate or inhibit the KCNQ1/KCNE1 channel. Some modulators, such as PUFA analogs [18,19,20] and chromanol 293B [21,22,23], are antiarrhythmic in that they can regulate the APD in cardiomyocytes and the QT interval in animals by modifying the KCNQ1/KCNE1 activation. In this review, we summarize the structural and biophysical properties as well as the regulation of cardiac KCNQ1/KCNE1 channels. 

## 2. KCNQ1/KCNE1 Channel Role in the Cardiac Action Potential

**Figure 1:**
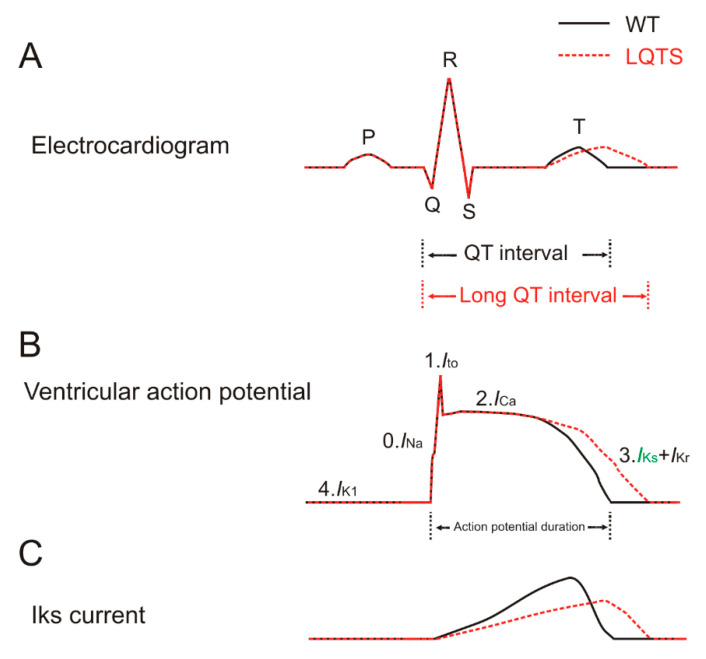
Long QT syndrome (LQTS) caused by a loss of the IKs current. Normal electrocardiogram, ventricular action potential and IKs current are depicted by black solid lines. The electrocardiogram, ventricular action potential and IKs current associated with LQTS are depicted by red dashed lines. Horizontal and vertical axes are not shown for clarity. (**A**) The electrocardiogram consists of five waves: P, Q, R, S and T waves. The P wave represents the depolarization of the atria. The QRS complex represents the rapid ventricular depolarization. The T wave represents the repolarization of the ventricles. The QT interval is the interval from the beginning of the Q wave to the end of the T wave. Patients with a prolonged QT interval can cause LQTS. Action potential duration is indicated. (**B**) The ventricular action potential consists of five phases, phase 0 to phase 4. Phase 0 is the depolarization phase that is mediated by an inward Na^+^ current. Phase 1 is mainly mediated by a transient outward K^+^ current. Phase 2 is the plateau phase that is mainly mediated by an inward Ca^2+^ current and outward K^+^ current. Phase 3 is the repolarization phase that is mainly mediated by outward K^+^ (IKs in green and IKr) currents. Phase 4 is mainly mediated by an outward K^+^ current (IK_1_). (**C**) The IKs current underlies the repolarization of the ventricular action potential, shown as phase 3 in (**B**).

The heart is a blood pump whose activity is controlled by cardiac electrical activity [1]. During each heartbeat, a healthy heart has an orderly progression of action potentials that start with the sinoatrial node, then spread out through the atrium, pass through the atrioventricular node down into the Purkinje fibers and finally spread out through the ventricles. The cardiac electrical activity can be detected by the electrocardiogram, also called ECG or EKG [2]. In a normal ECG recording, each heartbeat contains five different waves: P, Q, R, S and T waves (Figure 1A) [2]. The QT interval is measured from the beginning of the Q wave to the end of the T wave. Patients with a prolonged QT interval are likely to be clinically diagnosed as having long QT syndrome, which can lead to Torsades de pointes, ventricular fibrillation and sudden cardiac death [3,4,24]. Conversely, patients with a shortened QT interval might be diagnosed as having short QT syndrome (SQTS), which can lead to atrial fibrillation (AF) [3].

The ventricular cardiac action potential is mainly mediated by voltage-gated Na^+^, Ca^2+^ and K^+^ channels (Figure 1B) [3]. These channels are closed at the negative diastolic membrane potential but open upon membrane depolarization during systole. There are five phases of the ventricular action potential, phase 0 to phase 4 (Figure 1B) [1,3,25]. Phase 0 starts when the Nav channel activates, leading to the influx of Na^+^ ions from the outside of the membrane, which causes a strong depolarization. During phase 1, the Nav channel rapidly inactivates while a specific Kv channel activates, leading to a transient outward K^+^ current (I_to_). The inactivation of Nav and the activation of I_to_ lead to a small hyperpolarizing notch in the membrane potential. Phase 1 is followed by phase 2 when the Cav channel (L-type Ca^2+^ channel) activates. The current is now mainly mediated by the influx of Ca^2+^ ions, which contributes to the sustained depolarization or plateau phase of the ventricular action potential. Phase 3 begins when enough of the delayed rectifier Kv channels (IKs and IKr) are activated, causing a more outward K^+^ current than inward Ca^2+^ and Na^+^ currents. This process leads to the repolarization of the membrane potential and hence the termination of the action potential. The IKr and IKs contribute to the fast and slow components of the delayed rectifier K^+^ currents, respectively. A prolonged repolarization of the action potential caused by a loss of the IKs or IKr current is the most common cause for congenital LQTS-caused Torsades de pointes, ventricular fibrillation and sudden cardiac death (Figure 1C) [3,4]. For instance, loss-of-function mutations in IKs channels cause LQT1 (mutations in the KCNQ1 subunit) and LQT5 (mutations in the KCNE1 subunit) by reducing the amplitude of the repolarizing outward IKs current and thus increasing the APD [5,6]. Curiously, one KCNQ1 mutation was recently found to cause sever LQTS by reducing trafficking of IKr channels [26]. Conversely, rare severe gain-of-function mutations in IKs channels cause SQTS and AF by increasing IKs channel activity and thereby shortening the APD [3,27]. IKs channels have been shown as important for the repolarization process of both atrial and ventricular action potentials, but more important in the ventricular action potentials. Instead, the ultrarapid delayed rectifier IKur channel, only expressed in the human atria, is the predominant delayed rectifier current responsible for the atrial repolarization and is related to the most leading cause of AF [28]. In phase 4, the inwardly rectifying K^+^ (IK_1_) channel is open to set the diastolic membrane potential around −90 mV.

## 3. Architecture of KCNQ1/KCNE1 Channels

**Figure 2:**
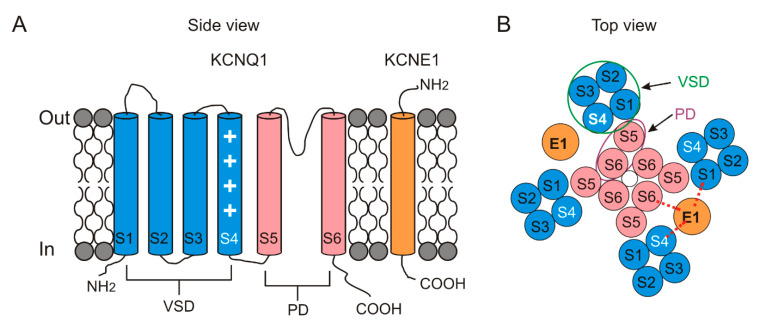
Topology of KCNQ1 and KCNE1. In the KCNQ1 subunit, S1–S4 transmembrane segments form the peripheral voltage-sensor domain (VSD) in blue and S5–S6 transmembrane segments form the central pore domain (PD) in pink. The KCNE1 subunit is one single transmembrane segment in orange. (**A**) Schematic side view of one KCNQ1 subunit and one KCNE1 subunit. The white plus symbol in S4 represents the positive gating charges. (**B**) Schematic top view of a tetrameric KCNQ1 channel with only two KCNE1 subunits. The number of the KCNE1 subunit varies from 1 to 4. KCNE1 is located in between S1, S4 and S6 from KCNQ1. Red dashed line indicates putative interactions between KCNE1 and S1, S4 and S6 from KCNQ1. A VSD (green circle) from one subunit is adjacent to a PD (purple oval) from its neighboring subunit.

The KCNQ1/KCNE1 channel consists of two subunits: the pore-forming subunit KCNQ1, also known as Kv7.1 or KvLQT1, and the auxiliary subunit KCNE1, also known as MinK [10,11] (Figure 2).

### 3.1. KCNQ1

**Figure 3:**
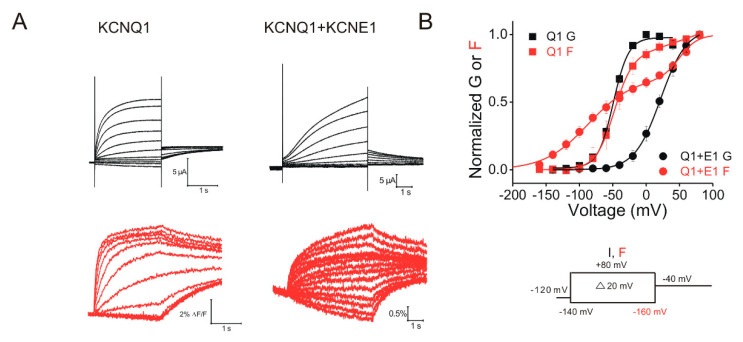
Voltage-clamp fluorometry recordings of KCNQ1 and KCNQ1/KCNE1 channels. (**A**) Representative current (black) and fluorescence (red) traces from KCNQ1 and KCNQ1/KCNE1 channels in response to the indicated voltage protocol (right). Cells are held at −120 mV and stepped to voltages between −140 (−160 mV for fluorescence) and +80 mV in +20 mV followed by a step to −40 mV. (**B**) Voltage dependence of currents (black) and fluorescence (red) from KCNQ1 (squares) and KCNQ1/KCNE1 (circles) channels. KCNE1 shifts the voltage dependence of the current activation of KCNQ1 to more positive voltages and separates the voltage sensors movement of KCNQ1. This suggests that S4 in KCNQ1/KCNE1 moves in two steps.

The KCNQ1 subunit itself expressed alone can form the tetrameric KCNQ1 channel (Figure 2B). KCNQ1 belongs to the family of KCNQ potassium channels, consisting of five members: KCNQ1–KCNQ5 [29]. The KCNQ1 channel is widely expressed in various tissues including the heart, inner ear, pancreas, kidney and brain [14,29,30,31,32]. KCNQ1 expressed alone elicits a fast activating current (relative to KCNQ1/KCNE1 currents, Figure 3A), undergoing a rapid inactivation, which can be seen in the hooked tail currents [33,34]. 

The KCNQ1 channel, like most other Kv channels, is composed of four subunits [14]. Each subunit contains six transmembrane segments, S1–S6 (Figure 2). The S1–S4 segments in each subunit form a peripheral voltage-sensing domain (VSD), while the S5 and S6 segments form a pore domain (PD). Four PDs together form the centrally located K^+^-conducting pore. The S4 segment harbors several positively charged residues and therefore senses the voltage changes across the membrane (Figure 2A). Like many Kv channels (but in contrast to IKr channels), KCNQ1 channels have a domain-swapped structure in which the VSD from one subunit is adjacent to the PD from the neighboring KCNQ1 subunit.

Ground-breaking high-resolution structures of many voltage-gated ion channels have been recently reported as advances have occurred in cryo-electron microscopy (cryo-EM) techniques. These structural studies have shed light on the structure–function relation of many ion channels. The *Xenopus* and human KCNQ1 structures in complex with Calmodulin (CaM) were revealed by MacKinnon’s lab using cryo-EM [35,36]. Generally, voltage sensors of most Kv channels are in the resting conformation at negative voltages while in the activated conformation at positive voltages [37]. However, some Kv channels, such as KCNQ1 [34,38], Shaker [39] and Kv1.2 [40], have been shown to also exhibit an intermediate conformation of voltage sensors [39,40,41]. Since structures of KCNQ1-CaM mentioned above were resolved at 0 mV, the voltage sensor of those channels was proposed to be in the activated conformation. To elucidate the voltage-sensing mechanism of KCNQ1 and likely other Kv channels, structures of voltage sensors at the intermediate conformation and resting conformation are needed. Recently, a nuclear magnetic resonance (NMR) structure of a human KCNQ1 VSD in an intermediate state [41] has been reported, although the PD is missing in this structure. This intermediate VSD conformation was suggested to be different from the cryo-EM activated VSD conformation using site-mutated mutagenesis and voltage-clamp fluorometry (VCF) [35]. Over the years, resolving the three-dimensional resting-state structure of VSD has been challenging in voltage-gated ion channels because the resting state predominates only at very negative voltages. Recently, Catterall and his colleagues [42] presented a cryo-EM structure of a bacterial Nav channel in the resting state. The VSD was captured in the resting state by introducing some voltage-shifting mutations and a disulfide crosslink (between G94C in S4 and Q150C in S5) to stabilize the resting state of S4. S4 was shown to move vertically ~11.5 Å with a significant rotation from the resting state to the activated state. Although this is the resting-state structure of a Nav channel in prokaryotes, it helps to understand how the voltage sensor moves and how it couples to the channel opening in voltage-gated ion channels. Maybe it will also be possible to reveal the resting VSD structure of KCNQ1 channels by using voltage-shifting mutations and disulfide crosslinks to trap the VSD in the resting state.

### 3.2. KCNE1

KCNE1 belongs to the family of KCNE auxiliary subunits. In total, there are five members of the KCNE family, labeled KCNE1–KCNE5. All of the members of the KCNE family are single-transmembrane segment proteins (Figure 2A) that differentially modify the properties of KCNQ channels in diverse tissues [14]. We here only focus on the KCNE1 modulation of KCNQ1 in the heart.

KCNE1 modifies the KCNQ1 channel function in several ways. For example, co-expression of KCNE1 slows the activation kinetics, increases the voltage-dependent current amplitude and shifts the voltage dependence of activation to more positive voltages of KCNQ1 channels in heterologous expression systems (Figure 3) [10,11]. This slowing of the activation kinetics is very important for generating the slowly activating IKs currents that regulate the APD and QT interval. Furthermore, some studies have shown that KCNE1 induces a larger single-channel conductance in KCNQ1 channels [43,44,45]. These studies suggested that the increase in the macroscopic current in the presence of KCNE1 is, at least partly, due to the increased apparent single-channel conductance of the KCNQ1 channel. KCNE1 has also been suggested to alter the ionic selectivity and to eliminate the inactivation of the KCNQ1 channel [33,46]. Conti et al. [46] found that, compared to KCNQ1 channels, KCNQ1/KCNE1 channels display a significantly lower Rb^+^/K^+^ permeability ratio. They further proposed that the Rb^+^/K^+^ permeability ratio is associated with the inactivation in KCNQ1 channels [47], yet the molecular mechanism underlying the relation between the Rb^+^/K^+^ ratio and the inactivation is not fully understood. 

The structure of KCNQ1/KCNE1 has not been obtained yet. However, a recent cryo-EM structure of the KCNQ1/KCNE3-CaM complex gives us a hint about how the KCNE1 subunit interacts with KCNQ1 and modulates the KCNQ1 activity [35]. Unlike KCNE1, KCNE3 dramatically shifts the voltage dependence of KCNQ1 channels to more negative voltages and thus makes KCNQ1/KCNE3 channels constitutively open in the physiological voltage range (from −90 to +50 mV) [48,49]. Comparing the structures between KCNQ1-CaM and KCNQ1/KCNE3-CaM, Mackinnon et al. [35] found that KCNE3 lies in between S1, S4, S5 and S6 from three different subunits, suggesting KCNE1 and KCNE3 might share a similar location when associated with KCNQ1. 

### 3.3. Interaction between KCNQ1 and KCNE1 

The stoichiometry of KCNQ1 and KCNE1 has been a long-lasting debate. Some studies have shown that KCNQ1 subunits and KCNE1 subunits together form the KCNQ1/KCNE1 channel with a flexible stoichiometry from 4:1 to 4:4 in heterologous expression systems [12,13]. Other evidence from experiments using single-molecule bleaching approaches indicates that the human surface KCNQ1/KCNE1 channel contains four KCNQ1 subunits and only two KCNE1 subunits (Figure 2B) [50]. The cryo-EM structure of KCNQ1/KCNE3 (a paralog of KCNE1) mentioned above supports the idea that a 4:4 stoichiometry is possible [35]. Therefore, the number of KCNE1 for the tetrameric KCNQ1 seems to be possible from one to four. Noteworthy, the KCNQ1/KCNE1 stoichiometry in cardiac cells has not been determined yet. In addition, other KCNE subunits are also expressed in cardiac tissues [51] and could compete with KCNE1 for association with KCNQ1, potentially making KCNQ1/KCNE1 stoichiometry more complex. 

How the KCNE1 subunit associates and functionally interacts with the KCNQ1 subunit remains unclear. The KCNE1 subunit has been suggested to be in direct physical contact with different sites of the KCNQ1 subunit (Figure 2B). Several studies [52,53,54] have demonstrated that KCNE1 directly binds to the pore, particularly the S6 segment, of the KCNQ1 channel to control the KCNQ1 gating. For example, FTL residues (F57, T58 and L59) in KCNE1 have been suggested to interact with S338, F339 and F340 in S6. Our recent study [49] about how KCNE1 acts on the gate of KCNQ1 supports the interaction between FTL residues and F339. In addition, the direct interaction between KCNE1 and S6 is supported by the evidence that a couple of residues (K41 and L42) from KCNE1 can form disulfide bonds with K324 in S6 when mutated to cysteines [55]. KCNE1 is also suggested to have contact with the S4 segment and shift the voltage dependence of the S4 movement [56]. Comparison of the S4 movement between KCNQ1 and KCNQ1/KCNE1 channels suggests that KCNE1 acts on S4 and separates the two components of S4 movements further in voltage dependence [49].

The extracellular end of the S1 segment has drawn some attention as an allosteric region of KCNQ1 gating. In this region, there is a short stretch of residues (positions 140–147) that when mutated are associated with cardiac arrhythmia [57]. Evidence from immunoblot and cysteine crosslinking experiments indicates that the extracellular end of KCNE1 makes state-dependent contact with the extracellular end of S1 in the KCNQ1 channel [55,57]. Tseng et al. [57] found that I145C could form disulfide bonds with KCNE1 G40C and K41C in a state-dependent way. After mutating, one at a time, the first four residues flanking the extracellular ends of S1 and KCNE1 to cysteines, Kass et al. [55] found that the disulfide bond can be formed between I145C and residue K41C and L42C. The direct contact between the extracellular end of S1 and KCNE1 is also supported by studies on two gain-of-function mutations (S140G and V141M) that cause short QT syndrome and atrial fibrillation. S140G and V141M greatly slow the KCNQ1/KCNE1 channel deactivation and hence increase the repolarizing K^+^ current in the action potential [58,59]. However, a big difference between these two adjacent mutations is that S140G slows the deactivation kinetics in the presence or absence of KCNE1, whereas V141M acts only in the presence of KCNE1. This suggests a direct interaction between V141 and KCNE1 [27] and also an indirect interaction between S140 and KCNE1 through the neighboring residue V141.

Taken together, several different sites, including S1, S4 and S6, of KCNQ1 could form disulfide bonds with residues 40–43 in KCNE1, suggesting that the extracellular end of KCNE1 is very flexible and engages in conformational changes during KCNQ1/KCNE1 association [27,52,55,56,57]. These electrophysiological studies are consistent with the idea that KCNE1 lies in between S1, S4 and S6 from different subunits of KCNQ1 (Figure 2B).

## 4. KCNQ1/KCNE1 Channel Activation by Voltage

### 4.1. Voltage Sensor Movement of KCNQ1/KCNE1 Channels

As a Kv channel, the KCNQ1/KCNE1 channel can be activated by membrane potential change. The positively charged S4 helix is the main voltage sensor of the voltage-sensing domain. It is widely accepted that the S4 helix senses the voltage changes across the membrane and moves outwards within the membrane. This outward movement leads to the conformational change of the gate via VSD–PD coupling, inducing the opening of the channel [15,16,17].

Most Kv channels can only open after all four independent voltage sensors move to their fully activated states [60]. However, our lab [61] has previously suggested that the KCNQ1 channel expressed alone can open after only one voltage sensor moves. Using VCF which simultaneously measures the S4 movement (by fluorescence) and channel opening (by ionic current), we [61] found that the kinetics and voltage dependence of the S4 movement are similar to those of channel opening in KCNQ1 channels (Figure 3B). The one-to-one relationship between the voltage sensor and channel opening suggests that the activation of one voltage sensor activation is enough to open the channel. We tested this idea by using the LQTS-associated mutation R231C that makes the KCNQ1 channel constitutively open by presumably locking the voltage sensor in the activated state [62]. We constructed a linked KCNQ1 dimer that contained one wt KCNQ1 subunit and one R231C subunit to generate tetrameric channels with two wt and two R231C subunits [63]. Compared to the wt-wt KCNQ1 dimer that displayed only 4% constitutive current, the wt-R231C KCNQ1 dimer displayed a much higher constitutive current of 29% at negative voltages. This suggests that KCNQ1 channels are 29% open with two activated (in the two R231C subunits) and two resting (in the two wt subunits) voltage sensors. Consequently, we proposed a ten-state allosteric model for KCNQ1 gating, where the open probability increases as more voltage sensors move to the activated state [63]. Taken together, in the absence of KCNE1, KCNQ1 channels can conduct with only one voltage sensor activation and the conductance seems to increase with each additional activated voltage sensor.

How many voltage sensors need to activate for KCNQ1 gate opening in the presence of KCNE1? Overall, two alternatives have been proposed by different groups [63,64,65,66]: (1) KCNQ1/KCNE1 channels can increase conductance by individual voltage sensor activation, or (2) KCNQ1/KCNE1 channels can only conduct when all four voltage sensors are activated. Recently, Westhoff et al. [65] found that KCNQ1/KCNE1 channels show detectable whole-cell and single-channel currents when one, two or three voltage sensors are restrained in the resting state by introducing the E160R mutation in KCNQ1 or F57W in KCNE1 in tandem constructs. E160R and F57W prevent the voltage sensor moving to the activated state, which is demonstrated by experiments conducting VCF and cysteine accessibility in the study. The authors, therefore, concluded that activation of all four voltage sensors is not required for the opening of KCNQ1/KCND1 channels. In support of the second possibility, we found that KCNE1 separates the two voltage sensor movements in the KCNQ1/KCNE1 channel by using VCF (Figure 3) and cysteines accessibility [66]. The first step shows the main rapid gating charge movement of the voltage sensor, which was demonstrated by the overlapped time and voltage dependence between the first fluorescence step and gating currents from KCNQ1/KCNE1 channels. The second step shows a slow movement of the voltage sensor, which corresponds with channel opening. According to our gating model for the KCNQ1/KCNE1 channels, at less depolarized voltages and at early times for more depolarized voltages, all four voltage sensors move fast and independently. Meanwhile, at more positive voltages, all four voltage sensors move with an additional slow and concerted step in order to open the KCNQ1/KCNE1 channel. Additionally, to test the second hypothesis that all four voltage sensors need to activate to open KCNQ1/KCNE1 channels, we used the mutation R231C that is assumed to lock the voltage sensor in the activated state [63]. KCNE1 association was found to abolish the constitutive currents of the wt-R231C dimer at negative voltages in KCNQ1 channels, as if KCNQ1/KCNE1 channels are not able to conduct with only two activated (R231C) voltage sensors. 

Voltage sensors in KCNQ1 channels have been shown to have resting, intermediate and activated conformations. Using VCF, Hou et al. [34] found that S4 activates in two steps: upon activation, S4 moves from the resting state to the intermediate activated state, and then to the fully activated state. The two-step movement of the voltage sensor in KCNQ1 channels is also observed in KCNQ1/KCNE1 channels in the study. This is in line with our previous study [66] that, in KCNQ1/KNCE1 channels, S4 activates in two steps (Figure 3B). We interpreted it as the first step involves the S4 gating charge movement and the second step involves a slow voltage sensor movement opening the channel. Comparing the NMR intermediate VSD structure to the cryo-EM activated VSD structure, S4 was found to move relative to the rest of the VSD with a ~5.4 Å translation of S4 toward the extracellular side from the intermediate state to the activated state [41]. Taken together, KCNQ1 and KCNQ1/KCNE1 channels are suggested to experience two steps of voltage sensor activation, where KCNQ1/KCNE1 channels only open after both steps, whereas KCNQ1 channels can open after the first step.

### 4.2. Gate Opening of KCNQ1/KCNE1 Channnels

The intracellular end of S6 from all four subunits has been shown to function as the activation gate. Most Kv channels are suggested to use a conserved glycine in S6 to act as a hinge for gate opening [67]. However, the corresponding residue in the KCNQ1/KCNE1 channel is alanine at the position of 336 [68]. Mutations of A336 alter the current amplitude and shift the voltage dependence of channel activation to more negative voltages in the KCNQ1 channel. Co-assembly of KCNQ1 with KCNE1 does not alter the effects of A336 mutations on the KCNQ1 channel, suggesting KCNE1 might have contacts with other regions to modulate the KCNQ1 gating [68]. On the other hand, previous structural and functional studies [69,70,71] have suggested that a highly conserved Pro-X-Pro motif, near the intracellular entrance of most Kv channels, is crucial for opening the gate. This motif is suggested to induce a kink in lower S6. Mutations of the proline or glycine in this sequence prevent the Shaker K^+^ channel from opening [71]. However, the corresponding motif in the KCNQ1 channel is a Pro-Ala-Gly motif. These motifs are also very important for the KCNQ1 gating [68]. According to the recent cryo-EM structure of KCNQ1-CaM, the lower S6 bends at the Pro-Ala-Gly motif. Consequently, the gate opening mechanism in the KCNQ1/KCNE1 channel seems different compared to other Kv channels and remains to be completely understood.

Another opening question is whether KCNQ1/KCNE1 and KCNQ1 channels share the common gating mechanisms or not. Since KCNQ1/KCNE1 and KCNQ1 channels have different single-channel conductance [43], pharmacology and ion selectivity [38], chances are that these two channels display different opening pore structures. This question may be solved when the KCNQ1/KCNE1 structure of an open state is determined.

### 4.3. VSD–PD Coupling of KCNQ1/KCNE1 Channels

In Kv channels, the voltage sensor movement triggers channel opening via the VSD–PD coupling, also known as electro-mechanical coupling [15]. For KCNQ1 expressed alone, Hou and his colleagues [34] suggested that S4 moves in two steps and that each step can open the channel: the S4 movement from the resting state to the intermediate activated state causes the intermediate-open state; the S4 movement from the intermediate state to the fully activated state causes the activated-open state. The intermediate-open state and activated-open state display a distinctive Rb^+^/K^+^ permeability ratio and XE991 (a KCNQ channel blocker) sensitivity [38]. More recently, using mutant cycle analysis and molecular simulations, they identified two groups of interactions that are highly crucial for the VSD–PD coupling when S4 is in the intermediate and activated states, respectively. In the intermediate S4 state, the C-terminal end of the S4-S5 linker interacts with the pore domain within the same subunit, which contributes to the canonical VSD-PD pathway [15]. On the other hand, in the activated S4 state, the N-terminal end of the S4-S5 linker interacts with the pore domain from the neighboring subunit. 

Similar to KCNQ1 channels, KCNQ1/KCNE1 channels were shown to have a two-step movement of the voltage sensor [34,66], yet KCNE1 association alters the VSD–PD coupling by suppressing the intermediate-open state such that KCNQ1/KCNE1 channels can only open when S4 is in the fully activated state. The molecular mechanism by which KCNE1 changes the VSD–PD coupling is unclear. However, our group has found that two atrial fibrillation-associated mutations (S140G and V141M) allow the KCNQ1/KCNE1 channel to open even when S4 helixes are in the intermediate states [27]. Both residues are in the extracellular end of the S1 helix. V141 was shown to directly crosslink with KCNE1, while S140 may indirectly interact with KCNE1 through its neighboring residue V141. This result suggests that these mutations could alter the VSD–PD coupling of KCNQ1/KCNE1 channels possibly by engaging with KCNE1. Consequently, we proposed a kinetic model wherein S140G and V141M affect the VSD–PD coupling and slow pore closing in the KCNQ1/KCNE1 channel, leading to increased KCNQ1/KCNE1 currents, SQTS and AF.

## 5. Physiological Modulators of KCNQ1/KCNE1 Channels

### 5.1. Protein Kinase A (PKA)

Cardiac KCNQ1/KCNE1 channels are regulated by sympathetic nervous stimulation via the activation of beta-adrenergic receptor-mediated PKA. During exercise or stress, stimulation of the sympathetic nervous system leads to a dramatically rapid heart rate. To allow the heart to have enough diastolic filling time between each heartbeat, a shortened ventricular action potential duration and a corresponding reduced QT interval in ECG recordings are necessary [72]. In patients with congenital LQTS, stimulation of sympathetic discharge during exercise increases the risk of tachyarrhythmias and sudden cardiac death [73,74]. The upregulated KCNQ1/KCNE1 channel activity via PKA activation was found to be important for regulating the cardiac action potential upon beta-adrenergic stimulation [72]. The activation of beta-adrenergic receptors increases the intracellular levels of cyclic adenosine monophosphate (cAMP) which in turn activates PKA. PKA activation then phosphorylates the KCNQ1/KCNE1 channel and enhances KCNQ1/KCNE1 function, therefore shortening the APD [72,75,76]. For example, Terrenoire et al. found [72] that PKA simulation speeds up the activation kinetics while slowing the deactivation kinetics of KCNQ1/KCNE1 channels in CHO cells. Yazawa and Kameyama [75] found that both isoprenaline (a beta-adrenergic receptor agonist) and PKA increase the amplitude of KCNQ1/KCNE1 currents in guinea pig cardiomyocytes. The regulation of KCNQ1/KCNE1 by phosphorylation at S27 in the KCNQ1 N-terminus requires protein phosphatase 1 (PP1) and the A-kinase anchoring protein Yotiao [77]. Yotiao was suggested to bind to the C-terminus in KCNQ1 via a leucine zipper (LZ) motif [77]. Mutation in the LZ motif disrupts the interaction between Yotiao and the LZ motif and thus leads to LQTS. Although most studies [72,75,77] have shown that upon beta-adrenergic simulation, PKA activation upregulates the activity of KCNQ1/KCNE1 channels, some groups [78,79] showed that sustained beta-adrenergic simulation downregulates the KCNQ1/KCNE1 activity in guinea pig cardiomyocytes. The downregulation of KCNQ1/KCNQ1 might be due to the reduced KCNE1 expression mediated by exchange protein directly activated by cAMP (Epac) but not PKA. Therefore, the effect of PKA activation following acute and chronic beta-adrenergic simulation on KCNQ1/KCNE1 channels might be different, and the molecular mechanism of the difference needs to be elucidated. Furthermore, whether PKA activation alters the voltage sensor movement and/or the VSD–PD coupling has not been tested yet, which may help to understand how PKA activation modulates the KCNQ1/KCNE1 function by beta-adrenergic simulation. 

### 5.2. Phosphatidylinositol 4,5-Bisphosphate (PIP_2_)

PIP_2_ is a phospholipid of the plasma membranes [80]. PIP2 was shown to activate different cardiac ion channels and transporters, while a depletion of PIP2 keeps channels and transporters inactive [80,81]. PIP2 regulation of ion channels has been suggested to keep these channels inactive during trafficking and processing of channels in intracellular membranes, which have low PIP2 levels [82]. In the heart, downregulation of PIP2 was suggested to prolong the ventricular action potential because cardiac KCNQ1/KCNE1 and hERG channels are sensitive to a lack of PIP2 [81]. According to previous structural and functional studies, PIP_2_ is a necessary cofactor for the KCNQ1/KCNE1 channel function and other KCNQ channels [35,83,84,85]. Loussouarn et al. [83] found that the intracellular application of PIP_2_ could significantly slow down the rundown of the KCNQ1/KCNE1 currents that spontaneously occurred in the excised patch-clamp recordings. The authors proposed a model in which PIP_2_ stabilizes the open state of KCNQ1/KCNE1 channels. Some LQTS-associated mutations (R539W and R555C) might weaken the interaction between KCNQ1/KCNE1 and PIP_2_ and therefore destabilize the PIP_2_-mediated open state of KCNQ1/KCNE1 channels [86]. Li et al. [84] found that KCNE1 increases the PIP_2_ sensitivity of KCNQ1 expressed alone about 100-fold, suggesting that KCNE1 is crucial for modulating the P1P_2_ sensitivity in KCNQ1 channels. Zaydman et al. [87] showed that PIP_2_ is required for the coupling between the voltage-sensing domain and the pore domain, such that without PIP_2_ the activation of the voltage sensor is not able to induce gate opening. This PIP_2_-dependent VSD–PD coupling was also seen in other KCNQ channels [88]. Using a mutation that abolishes the potentiation effect of PIP_2_ on the KCNQ channels, some studies [87,88,89] have identified a putative PIP_2_-binding pocket site that contains the C-terminus, A–B helix linker, S2-S3 linker and S4-S5 linker. The recent cryo-EM structure of the KCNQ1-CaM complex [35] supported the idea that in the absence of PIP_2_, the voltage sensor still moves but the pore remains closed. However, in the presence of PIP_2_, PIP_2_ binds to the loop connecting the S4-S5 linker and the C-terminus of KCNQ1 channels, which is consistent with the mutational studies above. Upon binding, PIP_2_ was seen to induce a large conformational change through a 180-degree rotation of CaM and thus to open the pore. The authors proposed that other members of the KCNQ family may share a similar PIP_2_-mediated gating mechanism, since the binding site harbors several conserved residues [35]. Recently, CP1, a molecule with some resemblance to PIP_2_, was shown to be able to substitute for PIP_2_ in the VSD–PD coupling of KCNQ channels [90]. CP1 was able to restore the prolonged APD induced by an IKr blocker back to normal in cardiomyocytes, which indicates CP1 could be a potential therapeutic for cardiac arrhythmias. 

### 5.3. Adenosine Triphosphate (ATP)

ATP is a major energy source in cardiomyocytes. The ATP level dramatically decreases in cardiac cells during heart failure and acute ischemia [91,92]. ATP can directly modulate the cardiac action potential and cause arrhythmias [93]. For example, elevated extracellular ATP was reported to trigger cardiac arrhythmias by prolonging the PR interval and partially blocking sinoatrial node activity and atrioventricular conduction in an isolated perfused rat heart [94]. In addition, in the electrically stimulated rat cardiomyocytes, increased extracellular ATP was shown to induce arrhythmias [95].

Intracellular ATP has been shown to regulate cardiac KCNQ1/KCNE1 activity. Loussouarn et al. [83] showed that the spontaneous rundown of KCNQ1/KCNE1 currents in the excised patch-clamp recordings could be slowed down by the addition of PIP_2_ and MgATP, which underscores the importance of ATP on channel opening. Li et al. [92] found that elevated intracellular ATP enhances the KCNQ1/KCNE1 activation in *Xenopus* oocytes and shortens the APD in cardiomyocytes. On the other hand, lowered intracellular ATP reduces the KCNQ1/KCNE1 activity and prolongs the APD. Using mutagenesis and VCF, ATP was shown to bind to the C terminus of the KCNQ1 channel and is required for the pore opening but not the voltage sensor activation or the VSD–PD coupling [92]. Some LQTS-associated mutations were shown to reduce the KCNQ1/KCNE1 activity by affecting the ATP sensitivity of KCNQ1/KCNE1 channels. Using simultaneous patch-clamp and FRET measurement, Kienitz and Vladimirova [96] found that a loss of ATP slowed the activation of KCNQ1/KCNE1 in Chinese hamster ovary (CHO) cells. In addition, since ATP depletion caused a more pronounced inhibition of KCNQ1/KCNE1 currents compared to PIP_2_ depletion, they proposed that intracellular ATP is a more potent modulator of KCNQ1/KCNE1 in comparison to PIP_2_. In KCNQ1/KCNE1 channels, the effects of PIP_2_ and ATP are independent of each other, although both of them are required to activate KCNQ1/KCNE1 channels. A previous study [92] suggested that PIP_2_ and ATP have different putative binding sites and activation mechanisms in KCNQ1 channels, as well as different KCNE1 dependence.

ATP also regulates other ion channels and therefore the action potential [97]. For example, activation of cardiac ATP-sensitive K (K_ATP_) channels shortens the action potential and causes arrhythmias, while suppression of K_ATP_ could prevent arrhythmias [98]. K_ATP_ channels activators were shown to protect the heart against ischemia and reperfusion arrhythmias [99,100]. Therefore, the K_ATP_ channel has been proposed as a target for anti-arrhythmic treatment [101]. Using patch-clamp, extracellular ATP inhibits the whole-cell current of ATP-sensitive K (K_ATP_) channels [97]. PIP2 was shown to prevent the current inhibition of K_ATP_ channels, suggesting the important role of PIP2 in the modulation of K_ATP_ channels by extracellular ATP. Since both cardiac KCNQ1/KCNE1 and K_ATP_ channels can be regulated by PIP2 and ATP and abnormal activity of these two channels can cause arrhythmias, maybe there is a connection, such as crosstalk, between them in the progression of cardiac diseases. Indeed, a decrease in the intracellular ATP reduces the APD by activating K_ATP_ channels while prolonging the APD by inhibiting KCNQ1/KCNE1 channels, suggesting that the heart has the ability to respond in different ways, maybe in different physiological and pathological conditions, to changes in PIP2 and ATP by various ion channel regulations.

## 6. Pharmacology of KCNQ1/KCNE1 Channels

### 6.1. Agonists 

To date, there have been a few known agonists for KCNQ1 and/or KCNQ1/KCNE1 channels, such as stilbenes [102,103], mefenamic acid [103,104], ML277 [105,106,107,108], phenylboronic acid (PBA) [109], zinc pyrithione [110], CP1 [90] and mallotoxin (MTX) [111].

Stilbene was one of the first activators of KCNQ1/KCNE1 to be studied years ago. Stilbenes have been shown to increase the current amplitude [102,103], slow the deactivation kinetics [102,103] and shift the voltage dependence of current activation to more negative voltages [103] of KCNQ1/KCNE1 channels expressed in *Xenopus* oocytes. The effects of stilbene on KCNQ1 expressed alone were also tested. Stilbene showed a significantly bigger activating effect in terms of amplitude, deactivation and conductance–voltage relation in the KCNQ1/KCNE1 channel compared to KCNQ1 alone. These differences suggest that KCNE1 is involved in the activation of KCNQ1/KCNE1 channels by stilbenes. Using site-directed and deletion mutants, stilbenes were suggested to bind to the extracellular end of KCNE1 and rescue the channel gating defect by mutations in this area, such as an LQT5-associated mutant D75N [103]. Mefenamic acid, a fenamate compound, has been found to shift the voltage dependence to a more negative voltage and slow down the deactivation kinetics [103]. Similar to stilbenes, mefenamic acid might bind to the extracellular residues flanking the transmembrane segment of KCNE1. In recent work, Wang et al. [104] found that mefenamic acid increases the open probability of KCNQ1/KCNE1 channels and that K41 in KCNE1 is required for mefenamic acid’s effect on KCNQ1/KCNE1 channels. The extracellular end of KCNE1 has been shown to be important for KCNQ1 and KCNE1 associations. 

In a high-throughput screen, Mattmann et al. [106] identified ML277 as a potent activator for the KCNQ1 channel and showed that ML277 is highly selective against other Kv channels including KCNQ2, KCNQ4 and hERG channels. This group [108] later found that ML277 potentiates heteromultimeric KCNQ1/KCNE1 channels but the increasing KCNE1 expression level reduced and eventually abolished ML277’s effect on KCNQ1/KCNE1 channels, indicating a competition between KCNE1 and ML277 when interacting with KCNQ1. In addition, ML277 was shown to shorten the APD in cultured human cardiomyocytes [108] and guinea pig ventricular myocytes [107], suggesting ML277 as a promising anti-arrhythmic drug. As previously reported [109], PBA activates the KCNQ1/KCNE1 channel by shifting the voltage dependence of current activation to more negative voltages. Although PBA was found to inhibit other Kv channels (Shaker and hERG channels), it activates other members of the KCNQ family (KCNQ1, KCNQ2/3 and KCNQ4). Consequently, PBA derivatives more selective for cardiac KCNQ1/KCNE1 channels can be potent activators for treatment of cardiac arrhythmias.

Interestingly, some common KCNQ2-5 activators have little or no effects on KCNQ1 or KCNQ1/KCNE1 channel activation. For example, retigabine, known as the first approved anti-epileptic drug, activates the KCNQ2-5 channels that are important for neuronal excitability [88,112,113]. Retigabine was shown to stabilize the open state of KCNQ2-5 channels by markedly shifting the voltage dependence of current activation to more negative voltages. However, KCNQ1 and KCNQ1/KCNE1 channels are retigabine-resistant [113] and the molecular mechanism is not fully clear. One possible explanation could be that KCNQ1 lacks the conserved Trp residue in other KCNQ channels that has been shown to be essential for the putative binding site for retigabine [88,114,115].

Taken together, although several KCNQ/KCNE1 activators have been reported, there are some limitations, including the low efficacy and the lack of specificity, that have to be overcome when thinking about the clinical use of these activators for the treatment of LQTS and cardiac arrhythmias.

### 6.2. Polyunsaturated Fatty Acid (PUFA)

**Figure 4:**
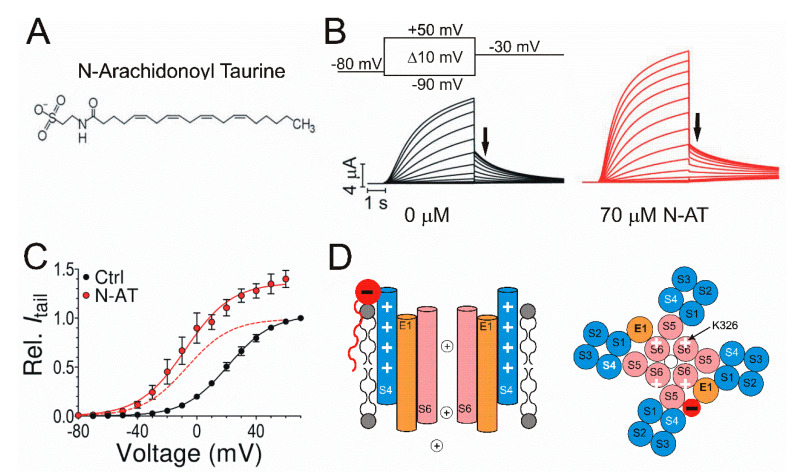
Effects of N-arachidonoyl taurine (N-AT) on cardiac KCNQ1/KCNE1 channels. (**A**) Structure of the polyunsaturated fatty acid (PUFA) analog N-AT with a negatively charged head group and a hydrophobic tail group. (**B**) Representative current traces from KCNQ1/KCNE1 channels in 0 μM N-AT (black) and 70 μM N-AT (red) in response to the indicated voltage protocol. Cells are held at −80 mV and stepped to voltages between −90 and +50 mV in +10 mV followed by a step to −30 mV. Arrows indicate the tail currents. (**C**) Voltage dependence of currents from KCNQ1/KCNE1 channels in 0 μM N-AT (black) and 70 μM N-AT (red) indicating an increased maximum conductance and negative shift of voltage dependence. Normalized voltage dependence of KCNQ1/KCNE1 channels in 70 μM N-AT is indicated as a red dashed line. (**D**) Illustration of the lipoelectric mechanism. Schematic side view (left) of KCNQ1/KCNE1 channels with S4 (blue), S6 (pink) and KCNE1 (orange). Electrostatic interaction between the negatively charged PUFA head group (red) and positive charges (white plus symbol) in S4. Schematic top view (right) of a tetrameric KCNQ1 channel with only two KCNE1 subunits. Electrostatic interaction between the negatively charged PUFA head group (red) and positively charged K326 (white plus symbol).

Recently, PUFAs have drawn more and more attention as they have been demonstrated to activate KCNQ1/KCNE1 channels efficiently, making PUFAs a promising approach for treating LQTS and cardiac arrhythmias. We have shown that PUFAs and their derivatives can enhance the activation of KCNQ1 and KCNQ1/KCNE1 channels by shifting the voltage dependence of current activation to more negative voltages and increasing the maximum conductance (Figure 4C) [18,116]. Furthermore, PUFA analogs were shown to have an antiarrhythmic effect on KCNQ1/KCNE1 currents by several pieces of evidence. One piece of evidence is that PUFA analogs are able to shorten the action potential duration and stabilize rhythmic action potential firing in isolated embryonic rat cardiomyocytes treated with Chromanol 293B, which prolongs action potentials and induces arrhythmic firing [18,117]. Another piece of evidence is that PUFA analogs can restore the QT interval and APD in isolated guinea pig heart perfused with the IKr blocker E4031 to induce a prolonged QT interval [18]. More recently, modified PUFAs were shown to shorten the QT interval in ex vivo and in vivo guinea pig hearts [118]. Finally, our group studied the effects of PUFA analogs on mutants that are associated with LQTS in KCNQ1/KCNE1 channels [19]. These LQTS-causing mutants are located in different sites of KCNQ1/KCNE1 channels and cause LQTS by distinctive mechanisms [119]. We demonstrated that N-arachidonoyl taurine (N-AT), a PUFA analog, restores gating, at least partly, in all these tested LQTS mutants, suggesting N-AT could be a novel KNCQ1/KCNE1 activator for LQTS treatment.

The molecular mechanism underlying the modulation of PUFAs and their analogs on the KCNQ1/KCNE1 function is fairly well understood. Structurally, PUFAs are amphipathic molecules that have both a charged hydrophilic head group and a hydrophobic tail group (Figure 4A) [120,121,122,123]. The negatively charged head group has been shown to be required for the Kv channel activation [18,123]. The negatively charged head group would electrostatically attract the positively charged S4 and enhance the S4 movement and the ensuing current activation (Figure 4D). Recently, we identified that a positively charged R231 in S4 is responsible for the electrostatic interaction between the head group and KCNQ1/KCNE1 channels [122]. The head group of PUFA was also suggested to electrostatically interact with the positively charged K326 in S6 to increase the maximum conductance of KCNQ1/KCNE1 channels by inducing a conformational change of the selectivity filter (Figure 4D) [122]. By testing PUFAs with different head groups, we found that PUFA analogs with taurine and cysteic head groups show the most pronounced activation of the KCNQ1/KCNE1 channel, suggesting that PUFAs may be developed for patients with different LQTS types [116].

Similar to the head group, the tail group of PUFA is necessary for the Kv channel activation [122,123]. PUFA would integrate into the plasma membrane by its hydrophobic tail group. By testing PUFAs with a carboxyl head group and different tail properties, Bohannon et al. [124] found that the position of the first double bond in the tail determines the PUFAs’ effect and binding affinity to the KCNQ1/KCNE1 channel.

PUFAs have been shown to modify not only KCNQ1/KCNE1 currents but also the Nav and Cav currents underlying the cardiac action potentials. Previous studies have found that PUFAs, such as 5,8,11,14,17-eicosapentaenoic acid (EPA), inhibit the Nav currents in cultured neonatal rat ventricular myocytes [125] and shorten the APD in isolated rat ventricular myocytes [126]. EPA was also found to suppress the L-type Cav currents in rat ventricular myocytes [127]. Therefore, PUFAs and PUFAs analogs are suggested to be antiarrhythmic in that they can activate KCNQ1/KCNE1 currents while inhibiting the Nav currents and Cav currents. We [20] recently found that PUFAs analogs influence the activity of cardiac KCNQ1/KCNE1, Nav and Cav channels via different mechanisms. In addition, by testing PUFA analogs with different head group and with tail group properties, different PUFAs analogs display different selectivities for KCNQ1/KCNE1, Nav and Cav channels. PUFA analogs that are more selective for the KCNQ1/KCNE1 channel compared to Nav and Cav channels are able to shorten a prolonged action potential in simulated cardiomyocytes without altering other properties of the action potential. Collectively, PUFA, present in fish oil, and its analog are antiarrhythmic and potential candidates for the treatment of LQTS and cardiac arrhythmias.

### 6.3. Antagonist

The development of a selective KCNQ1/KCNE1 blocker is of great importance for the design of potential antiarrhythmic strategies. Several antagonists of KCNQ1/KCNE1 channels have been reported including Tetraethylammonium (TEA) ions [128,129,130], Chromanol 293B [21,22,131], benzodiazepine L7 [132], HMR 1556 [133,134], JNJ-303 [135,136], UCL2077 [137], XE991 [38,41,138], amitriptyline [139], Tricyclodecan-9-yl-xanthogenate (D609) [140] and insulin [141]. Here, we only focus on a general Kv channel blocker, TEA, and a selective KCNQ1/KCNE1 channel blocker, Chromanol 293B.

The organic ion TEA has long been known to block Kv channels [142,143,144] including KCNQ1/KCNE1 channels that can be inhibited by internal TEA and inhibited weakly by external TEA [129,130]. Kurokawa et al. [130] found that external TEA rapidly and reversibly blocks both the KCNQ1 and KCNQ1/KCNE1 channels expressed in CHO cells. As an open channel blocker, TEA was suggested to bind to the extracellular loop of the outer pore of the KCNQ1/KCNE1 channel, which is consistent with the common TEA-binding site in other Kv channels [143,144]. By testing the TEA blockade effect on KCNQ1-4 channels in CHO cells, Hadley et al. [145] found that KCNQ2 has the most robust sensitivity, KCNQ1 and KCNQ4 have intermediate sensitivity and KCNQ3 has little sensitivity, to external TEA. The differential sensitivity to TEA might be due to the tyrosine residue of the outer pore in KCNQ2 but lacking in other KCNQ channels that has been shown to be responsible for TEA binding in Kv channels [144]. In addition, internal TEA was found to inhibit KCNQ1 in the presence or absence of KCNE1 expressed in *Xenopus* oocytes [128]. Internal TEA binds to the intracellular pore and blocks the potassium current, which is the canonical pore occlusion mechanism. This blockage mechanism has been seen in other Kv channels [143,144]. However, KCNQ1/KCNE1 channels, compared to KCNQ1 channels, were shown to be more sensitive to internal TEA, suggesting KCNE1 helps determine the KCNQ1 pharmacological properties.

Chromanol 293B has been widely used as a specific KCNQ1/KCNE1 channel blocker and has been proposed as a potential class III antiarrhythmic agent. A class III antiarrhythmic agent acts by lengthening the repolarization phase of the cardiac action potential and causes a concomitant increase in the effective refractory period at slower heart rates [23]. Chromanol 293B was shown to block the guinea pig KCNQ1/KCNE1 channels expressed in *Xenopus* oocytes and the KCNQ1/KCNE1 current in guinea pig cardiomyocytes [22]. Chromanol 293B showed little effect on the IKr current which contributes to the depolarization of the cardiac action potential together with the KCNQ1/KCNE1 current in guinea pig cardiomyocytes [22]. In addition, it also exhibited no inhibitory effect on the cardiac hERG current expressed in *Xenopus* oocytes [22]. Bosch and his colleagues [21] tested the effects of Chromanol 293B on the Kv, Nav and Cav currents and action potential in human and guinea pig cardiomyocytes. Chromanol 293B inhibits KCNQ1/KCNE1 but no other Kv (I_K1_ and I_to_), Nav and Cav currents. It also prolongs the action potential duration in cardiomyocytes. These results together suggest that Chromanol 293B is a rather selective blocker for KCNQ1/KCNE1 channels. A previous study [131] suggested that Chromanol 293B binds to KCNQ1 through electrostatic interaction with a potassium ion in the selectivity filter of the channel. HMR 1556, a chromanol derivative, has been shown to block the KCNQ1/KCNE1 channel with a higher binding affinity, compared with Chromanol 293B [133].

## 7. KCNQ1/KCNE1 Channel as a Target for Long QT Syndrome Treatment

The most common clinical treatment of LQTS over the years has been to use beta-blockers which reduce pro-arrhythmic sympathetic activity [4,5]. Beta-blockers were found to be effective for patients with LQT1 and maybe LQT2 [4]. Nevertheless, some patients do not tolerate beta-blockers. For those who have LQT3, taking sodium channel blockers, such as flecainide, has been suggested as beneficial [146]. Another useful therapy is to surgically implant cardioverter defibrillators [147]. These medical devices help to monitor and restore the heart rhythms and may be appropriate in some patients. Other treatment options include potassium supplementation [148] and sympathetic denervation (such as left cardiac sympathetic denervation for the treatment of LQTS and catecholaminergic polymorphic ventricular tachycardia) [149,150]. Thus far, current treatments for LQTS do not restore the QT interval to normal or cannot be applied to all individual patients, although they greatly improve patients’ chances of survival [5]. In addition, since no direct or personalized therapeutics have been developed to target the channelopathies that lead to LQTS, particularly important is understanding the structure–function relationship of ion channels (such as KCNQ1/KCNE1 channels) and the drug–channel interaction (such as the activation of KCNQ1/KCNE1 by PUFA and PUFA analogs). In addition, in a recent simulation study, activators of KCNQ1/KCNE1 channels were proposed as the safest strategy for the development of LQTS treatments [7]. On the other hand, development of selective blockade of KCNQ1/KCNE1 channels has been studied as a strategy for providing more effective class III antiarrhythmic agents [23,151].

## 8. Conclusions

The KCNQ1/KCNE1 channel belongs to the superfamily of voltage-gated K channels that are critical for cardiac excitability. In the heart, the KCNQ1/KCNE1 channel regulates the ventricular action potential duration by contributing to the repolarization phase of the ventricular action potential. Dysfunctional KCNQ1/KCNE1 channels prolong the APD and cause life-threatening LQTS and cardiac arrhythmias. Although several activators or inhibitors have shown to modulate KCNQ1/KCNE1 channels and regulate the APD, no modulator is strictly selective for KCNQ1/KCNE1 channels. Note that LQTS is associated with over 300 mutations found in KCNQ1/KCNE1 channels and no personalized treatment for every individual is available. PUFA analogs have shown encouraging antiarrhythmic effects in cardiomyocytes and animal models by modifying the activation of the KCNQ1/KCNE1 channel through different mechanisms. Considering the efficacy and commercial availability of PUFA analogs, future studies will further test the potential of PUFA analogs as therapeutics in cardiac arrhythmias. Furthermore, the rich diversity and flexibility of PUFA analogs by designing different head or tail groups would be of great use to develop more individualized treatments for LQTS patients with distinctive phenotypes.

The KCNQ1/KCNE1 channel contains both a KCNQ1 subunit and a KCNE1 subunit. KCNE1 modifies the KCNQ1 channel in many ways including the current amplitude, activation kinetics, single-channel conductance, voltage sensor movement, VSD–PD coupling and ion selectivity as well as drug sensitivity. In addition, KCNE1, as a single transmembrane domain, has been suggested to interact with several different transmembrane domains of KCNQ1 and with different stoichiometry. The molecular mechanism by which KCNE1 alters KCNQ1 properties still remains unknown, although many models have been proposed using electrophysiology, biochemistry, fluorescence spectroscopy and simulations. This one big open question suggests that there are more exciting studies and experiments of KCNQ1/KCNE1 channels to be conducted.

The three-dimensional cryo-EM structures of KCNQ1-CaM, KCNQ1/KCNE3-CaM and KCNQ2 channels have helped to explain some functional studies of KCNQ channels with or without different KCNE subunits. However, the lack of a cryo-EM structure of the KCNQ1/KCNE1 channel still is a barrier to fully understand how the KCNQ1/KCNE1 channel functions in health and disease, as well as how drugs bind to and modulate the channel. Revealing the resting-state and the intermediate-state structures of KCNQ1/KCNE1 would be helpful to understand the voltage-sensing mechanism of KCNQ1/KCNE1 channels as KCNQ1/KCNE1 is suggested to display two-step voltage sensor movement, which is different from most Kv channels.

## Abbreviations

LQTS	Long QT syndrome
IKs	The slowly activating delayed-rectifier K^+^ current
Kv	Voltage-gated potassium channel
Nav	Voltage-gated sodium channel
Cav	Voltage-gated calcium channel
VSD	Voltage-sensing domain
Na^+^	Sodium ions
K^+^	Potassium ions
Ca^2+^	Calcium ions
PD	Pore domain
PUFA	Polyunsaturated fatty acid
ECG	Electrocardiogram
SQTS	Short QT syndrome
IKr	The rapidly activating delayed-rectifier K^+^ current
APD	Action potential duration
Rb^+^	Rubidium ions
CaM	Calmodulin
PIP_2_	Phosphatidylinositol 4,5-bisphosphate
VCF	Voltage-clamp fluorometry
Cryo-EM	Cryo-electron microscopy
NMR	Nuclear magnetic resonance
HCN	Hyperpolarization-activated and cyclic nucleotide-gated channel
PKA	Protein kinase A
cAMP	Cyclic adenosine monophosphate
PP1	Protein phosphatase 1
LZ	Leucine zipper
Epac	Exchange protein directly activated by cAMP
ATP	Adenosine triphosphate
K_ATP_	ATP-sensitive K channel
FRET	Fluorescence resonance energy transfer
MTX	Mallotoxin
PBA	Phenylboronic acid
N-AT	N-arachidonoyl taurine
EPA	5,8,11,14,17-eicosapentaenoic acid
TEA	Tetraethylammonium ions
CHO	Chinese hamster ovary
UCL2077	3-(triphenylmethylaminomethyl)pyridine
XE991	10,10-Bis(4-pyridinylmethyl)-9(10H)-anthracenone dihydrochloride
IKur	The ultrarapid delayed-rectifier K^+^ current
AF	Atrial fibrillation
